# Normal Gaze Cueing in Children with Autism Is Disrupted by Simultaneous Speech Utterances in “Live” Face-to-Face Interactions

**DOI:** 10.1155/2011/545964

**Published:** 2011-11-24

**Authors:** Douglas D. Potter, Simon Webster

**Affiliations:** ^1^School of Psychology, University of Dundee, Dundee DD1 4HN, UK; ^2^Your Voice, Unit 15, Coatbridge Business Centre, 204 Main Street, Coatbridge ML5 3RB, UK

## Abstract

Gaze cueing was assessed in children with autism and in typically developing children, using a computer-controlled “live” face-to-face procedure. Sensitivity to gaze direction was assessed using a Posner cuing paradigm. Both static and dynamic directional gaze cues were used. Consistent with many previous studies, using photographic and cartoon faces, gaze cueing was present in children with autism and was not developmentally delayed. However, in the same children, gaze cueing was abolished when a mouth movement occurred at the same time as the gaze cue. In contrast, typical children were able to use gaze cues in all conditions. The findings indicate that gaze cueing develops successfully in some children with autism but that their attention is disrupted by speech utterances. Their ability to learn to read nonverbal emotional and intentional signals provided by the eyes may therefore be significantly impaired. This may indicate a problem with cross-modal attention control or an abnormal sensitivity to peripheral motion in general or the mouth region in particular.

## 1. Introduction

Gaze-following behaviour is central to the Mindblindness model of social cognitive development in autism [1]. This innate modular model predicts that when gaze following occurs in autism it will be typical. When gaze following occurs in autism at a level below that expected for the child's chronological age, gaze following will be delayed but otherwise typical.

An alternate possibility exists, not predicted by this model. Gaze following may develop successfully but atypically in autism. This is the atypical modularisation hypothesis. It is normal to process information from the mouth when someone is speaking [2] while simultaneously processing information from the eyes about the speaker's direction of attention and intentions. This is consistent with some current theories of attention which allow simultaneous processing of information at more than one location (e.g., [3, 4]). When typical children attend to a face, they tend to look more at the eyes than at the mouth (e.g., [5]), and this bias is evident from early infancy [6]. In autism, there is a bias to look more at the mouth than at the eyes in childhood [5, 7]. However the mouth provides both visual and auditory cues, and people with autism may pay undue attention to the mouth because of limitations in “central coherence” [8]. This may result in an inability to balance the interaction between those aspects of perceptual organisation which maintain the experience of the perceptual whole while allowing more detailed processing of motivationally relevant component features. 

Farroni et al. [9] have demonstrated that humans are sensitive to eye contact from birth. In previous research related to the present study we have reported evidence of atypical development in the use of gaze information. In a study of “live” face-to-face eye gaze direction detection, older children with autism and typical children were equally accurate at determining the direction of gaze while younger children with autism were impaired relative to a matched control group [10]. In a further study using photographic images we measured ability to detect direct eye contact and sensitivity to gaze shifts of 5 to 20 degrees in steps of 5 degrees. In both tasks younger children with autism showed a greater impairment in performance than older children [11]. The research above is consistent with anecdotal evidence of abnormal utilisation of gaze information and the apparent development of alternative strategies to compensate for this impairment. The research described in the next section explored the possible role of abnormal attention control as a basis for the impairments described above. 

Many children with autism develop gaze following (e.g., [12]), but they may develop this skill despite a relative insensitivity to social information from other people's eyes. In a recent review of eye gaze and attention cueing research in children diagnosed with autism, Nation and Penny [13] concluded that the majority of evidence accumulated, using gaze cues in Posner cueing paradigms, suggests that autistic individuals produce normal reflexive shifts of attention. To the extent that there is variability in these findings, this may result from underlying differences in sensitivity to eye gaze cues in the general population such as gender, presence of autistic traits, or anxiety [14]. The challenge lies in determining whether this “normal” performance utilises attention control mechanisms that are influenced by the social significance of these stimuli. Ristic et al. [15] provide an important piece of evidence regarding the social significance of cues. Static schematic faces were used to provide cues in a Posner paradigm. If the cues were 80% valid then both normal and autistic children showed typical cuing effects. However, if the cues were 50% valid and therefore not predictive then normal children still showed cuing effects while autistic children did not. This suggests that normal children were unable to override the effect of social significance as a cue to shift attention. 

Some of the issues raised regarding variability of findings cited above were addressed in a more recent study by Pruett et al. [16]. They systematically explored social and nonsocial cueing of visuospatial attention in autistic and typically developed children and adults using 50% and 80% valid gaze, box and arrow cue conditions in a Posner paradigm. In their first experiment, in a large group of typical adults, they demonstrated classic interactions between “exogenous” (box) and “endogenous” (arrow, gaze) cueing effects. They interpreted a lack of difference between arrow and gaze cuing at 50% and 80% validity as consistent with both cues activating the same attention control mechanisms, though perhaps by different routes. In their second experiment with typical male and female children and adults they observed no sex differences but adults were better at the task than children. In their third study of a group of 27 autistic spectrum disorder (ASD) and 25 matched typical children, the ASD children were slower in the task but the cuing effects were not significantly different. 

It would appear that abnormal eye gaze behaviour in children diagnosed with autism does not result from fundamental differences in basic attention control mechanisms. However, it is possible that the experimental situations used in previous studies may be too artificial to demonstrate effects that are evident in real world interactions. In particular, many gaze cues occur in the context of speech output. The first aim of this study was therefore to explore the possibility that autistics have a tendency to orient to speech and mouth movement in preference to the eyes and that this would disrupt their otherwise normal gaze cued behaviour. The second aim of this study was therefore to provide a baseline for this experiment by determining if the previously observed dynamic and static cuing effects are replicated in ASD children in “live” face-to-face interactions. 

The present study included three conditions. The first condition (with eye motion) tested for gaze cueing in both groups. A second condition, without eye motion, assessed whether gaze cueing occurred in response to static, averted eye-gaze cues. In typical infants, gaze-following occurs in response to moving eye-gaze cues but not in response to static cues [17, 18]. If children with autism behaved in the same way as infants, this would imply that their gaze-following competence was developmentally delayed. In the third condition, the experimenter said a monosyllabic word at the same time as he made a gaze shift to the left or right to test whether a speech utterance would disrupt gaze cueing in children with autism. Participants were a group of children with autism and a closely matched group of typical children.

## 2. Method

### 2.1. Participants

19 children with autism and 16 typically developing control participants took part in this study. Participants who were included in the analysis had prorated WISC-R IQs above 75 and scored within the appropriate range on the parent-completed autism screening questionnaire (ASQ: [19]). Participants in the autism group all had existing diagnoses of autism, autism spectrum disorder, or Asperger syndrome. This information was collected on a parental consent form. In the autism group, 2 participants were excluded because their ASQs were not returned by parents, and 1 was excluded because his ASQ score was below cutoff. 6 participants were removed to permit group matching on four variables (chronological age, vocabulary scaled scores, picture completion scaled scores, and prorated IQ from the WISC-R). Two participants with autism did not complete one condition due to technical problems. In total, 8 participants with autism were included in the analysis. In the control group, 3 participants' ASQs were not returned, and a further 4 control participants were excluded from the analysis to allow matching on all four variables. In total, 9 typically developing control participants were included in the analysis. The autism and control groups were closely matched (all *P* ≥ .49: see [20]) on age, vocabulary scaled score, picture completion scaled score, and prorated IQ (Table 1).

### 2.2. Apparatus

Time constraints required prorating, but IQ subtests were selected carefully. A verbal and nonverbal WISC-R dyad was selected to give scores that would correlate highly with full scale IQ (FSIQ; *r* = .87: [21]). As a nonverbal test, Block Design would typically produce higher correlations with FSIQ, but this test is a peak of ability in autism. Close group matching on verbal and nonverbal ability and on prorated FSIQ was used in order to minimise the effects of any verbal/nonverbal performance discrepancies in the autism group. 

The experiment was run on a laptop computer using DMDX experimental software developed at Monash University and at the University of Arizona by K. I. Forster and J. C. Forster. Participants responded using a PC gamepad. Reaction times were recorded with millisecond accuracy. The participant and the experimenter sat at opposite ends of a table that was 35 inches (90 cm) in length. The participant and the experimenter each used a chinrest that was clamped to their end of the table. The laptop sat immediately in front of the experimenter. The experimenter's hands rested on a small board that sat on top of the laptop's keyboard. The laptop's screen was tilted down towards the experimenter at an angle of 45 degrees such that the experimenter's hands were hidden from the participant's view. 

On either side of the experimenter's chinrest was a thin metal pole, and on each pole at the experimenter's eye level there was a red light emitting diode (LED) of 3 mm diameter. Two small touch-sensitive switches were positioned on top of a small board behind the laptop's screen. With this arrangement the experimenter was able to press either switch to illuminate the left or right LED, or the space bar on the laptop's keyboard, without the participant seeing any hand movements. 

The experiment was computer controlled. The experimental software provided auditory cues to the participant and to the experimenter. The participant heard cues through a PC speaker that was directed towards the participant. The experimenter heard cues through in-ear earphones. The participant's cues were recorded on the audio track of a digital video camera. The video camera also recorded the visual cues that the experimenter produced (eye movements and LED illuminations). The video camera was mounted on a tripod beside the table and had a field of view that included the experimenter's face and the LEDs on either side of the experimenter's face.

### 2.3. Stimuli

In all conditions, each trial began with a 21 ms auditory tone. This was followed by a period of approximately 900 ms of eye contact from the experimenter. However, in the condition without eye motion, the experimenter's eyelids closed after 300 ms eye contact whilst he moved his eyes towards the target. At 900 ms after the start of the trial the experimenter produced a gaze shift (or opened their eyes to provide a gaze cue without eye motion) to the left or to the right. Approximately 300 ms after this gaze cue, one of the LEDs was illuminated by the experimenter on the left or on the right. 

In the condition with eye and mouth motion, all spoken words began with one of four plosive stops: b, d, p, or t. There were 15 words each starting with b, d, p, or t. Words were selected from the 3,000 most frequent words in English as reported in the Longman Dictionary of Contemporary English (2003 edition). The distribution of stimulus words was balanced across trial types and between the first and second halves of the task.

### 2.4. Design

The order of presentation of conditions was randomised. Each condition consisted of 64 trials. 4 practice trials were followed by 2 blocks of 30 trials. There was a brief break between blocks. The order of trials was randomised within blocks. The quantity of each type of trial was balanced within each block. The within-group factor was cue validity (valid or invalid).

### 2.5. Experimental Procedure

The participant was shown the two targets (the LEDs). They were told that they would hear a tone through the speaker at the start of each trial. They were instructed to look towards the experimenter's eyes when they heard the tone. They were told that the experimenter would look towards their eyes at the same time and that one of the targets would then light up. If the target on the left lit up, the participant was to press a button on the left of the gamepad as quickly as possible. If the target on the right lit up, they were to press a button on the right. Participants were not told that the experimenter would make gaze shifts. Instead, they were told that the experimenter's eyes would give them no information about which target would light up. They were informed that there would be several short breaks during the task and they were then reminded that the experimenter's eyes would give them no useful information. Participants were told that they could stop the experiment at any time. 

The experimenter (S. Webster) began each trial by pressing the space bar on the keyboard. This triggered auditory instructions which only the experimenter heard through his earphones: “left left”, “left right”, “right left,” or “right right”. The first word told the experimenter the direction in which he should make a gaze shift. The second word told him which LED he should switch on. Before the start of each trial, the experimenter looked at a point just above the participant's head. Using peripheral vision, the experimenter checked that the participant was attending (generally) in the experimenter's direction. To begin each stimulus sequence, the experimenter again pressed the space bar on the keyboard. This triggered the onset of the auditory cues. The first tone was heard simultaneously by the experimenter (through earphones) and by the participant (through a speaker). This tone signalled to the participant that they should look towards the experimenter's eyes, and to the experimenter that he should make eye contact, immediately after the tone. 

The next tones were heard only by the experimenter. The second tone occurred 300 ms after the start of the trial, and subsequent tones were equally spaced: each tone lasted 21 ms and was followed by 280 ms of silence. The intertone interval was therefore 300 ms. During each trial, the second and third tones allowed the experimenter to anticipate when the (significant) fourth and fifth tones would occur. On the fifth (final) tone, the experimenter pressed a switch to illuminate the appropriate LED. The experimenter held this switch down until he heard the participant releasing a button on the gamepad. To begin the next trial, the experimenter again pressed the space bar on the keyboard. 

In the condition with eye motion, the experimenter made a gaze shift in the appropriate direction on the fourth tone (approximately 900 ms after the start of eye contact). In the condition without eye motion, the experimenter closed his eyes on the second tone. On the third tone, the experimenter moved his eyes (beneath his eyelids) to the left or right. On the fourth tone, the experimenter opened his eyes. In the condition with eye and mouth motion, the experimenter heard a monosyllabic word before each trial and then produced this word at the same time when he made a gaze shift (on the fourth tone).

### 2.6. Procedure for Analysis of Raw Data

The analysis of data from this study was complex, so the experimenter coded all trials.

For each trial, the participant's reaction times (recorded by the laptop) had to be synchronised with the onset of the experimenter's gaze cues and with the (manual) onset of the LED. The onset of the first tone was taken to be the beginning of the trial. Video analysis was used to time each event (e.g., tone onset). The video frame duration was 40 ms (PAL system), so the measurement error was ±20 ms for video data. Because of this measurement error, the median of the experimenter's cue-target durations was used instead of the mean. The mean of median cue-target durations was later calculated for each group (Table 2) to permit analysis by ANOVA. 

During analysis of the video data, experimenter errors were noted. In subsequent analyses these trials were discarded. Errors included looking in the wrong direction, pressing the wrong LED switch, smiling, and blinking during a trial. Blink trials were defined as trials on which the eyelids covered more than half of the eye. In order to ensure that the experimenter's gaze cues were of consistent duration, the durations of these cues were determined from video data. The cue-target interval was the time between the onset of the gaze shift and the onset of the target LED. 

An adjusted reaction time was calculated for each trial for each participant. Participants' reaction times were recorded (by the laptop, using DMDX software) from the onset of the first tone. The onset time of the target LED was variable because the experimenter pressed a button to illuminate the LED on each trial. The participant's reaction time (on each trial) was defined as the time from target (LED) onset to the time of the participant's response. This value was calculated as the time from the onset of the target LED (as measured from video camera data) to the start of reaction time recording, plus the participant's recorded reaction time. The time of onset of the target was calculated as the (1246 ms) period between the first tone and the start of reaction time recording, minus the time between the first tone onset, and the target onset (from video data). These calculations gave a reaction time for each participant, for each trial, that was equivalent to the time from the onset of the LED to the time of their response. In the condition with eye motion, the events of interest were: tone onset, gaze onset, and target onset. “Gaze onset” was the time of onset of the gaze shift cue.

In the condition without eye motion, the events of interest were: tone onset, eyes closed, eyes open (start), eyes open (end), and target onset. “Eyes closed” was the time of the first frame in which the experimenter's eyes were fully closed, that is, in which the white of his eye was no longer visible. “Eyes open (start)” was the time of the first frame in which the white of the experimenter's eye was again visible. “Eyes open (end)” was the time of the frame in which the eyes were fully open. This was taken to be the second of two consecutive frames in which the position of the eyelid and eyelashes appeared to be identical. 

There was a specific type of cue error for the condition without eye motion: motion errors. Trials were marked as “motion” trials when lateral motion of the eye was evident over more than one frame after opening of the eyes. These trials were not included in further analyses. For this condition, the onset of the gaze cue (“gaze onset”) was defined as the midpoint between the start of eye opening and end of eye opening. The cue-target interval was therefore the time of onset of the target LED, minus the time of the midpoint between the start and end of eye opening. Consequently, cue-target intervals were short in this condition. 

In the condition with eye and mouth motion, the events of interest were: tone onset, mouth motion onset, gaze onset, and target onset. Mouth motion was defined as compression or expansion of the lips occurring after the onset of the tone that marked the beginning of the trial.

## 3. Results

### 3.1. Errors

Overall, 3.9% of trials were dropped from analyses due to experimenter or participant errors (defined above). The proportion of errors that the experimenter made in each condition is shown in Table 2. In all conditions, there was no significant difference in the amount of errors made by the experimenter for the autism and control groups (see Table 2). In the condition without eye motion, the experimenter made errors on 7.6% of trials. This was mostly due to motion errors (5.8% of trials). The proportion of errors that participants made in each condition is reported in Table 3. In all conditions, there was no significant difference in the amount of errors made by the autism or control groups (see Table 3).

### 3.2. Analysis of the Experimenter's Cues

In all conditions, there were no significant correlations between the experimenter's cue durations and participants' reaction times for any trial type (all *P* ≥ .11, mostly *P* > .40). The analysis therefore reports effects of cue validity rather than any effects of cue durations on participants' reaction times. In the condition with eye and mouth motion, mouth motion began 158 ms before gaze shift onset for the autism group and 131 ms before gaze shift onset for the typical control group. A two-tailed *t*-test confirmed that the difference between these mean durations was not significant (*t*(15) = 1.127, *P* = .28).

### 3.3. Analysis of Participants' Responses

Participant's reaction times are given in Table 4. In all conditions, participants' reaction times were strongly intercorrelated (all *P* ≤ .001). There were therefore no anomalous effects of trial type on participants' response times. For each condition, a  2 × 2 ANOVA was performed on participants' reaction time data using factors of cue validity (valid or invalid) and participant group (autism or control). In the condition with eye motion only, cue validity significantly improved reaction times (*F*[1,15] = 23.947, *P* < .001) but there was no significant difference between groups (*F*[1,15] = .032, *P* = .86). There was no significant interaction between cue validity and participant group (*F*[1,15] = 3.017, *P* = .10). In the condition without eye motion, cue validity again significantly improved reaction times (*F*[1,15] = 15.332, *P* = .001); groups were again not significantly different (*F*[1,15] = 1.103, *P* = .31). The interaction between cue validity and participant group was also not significant (*F*[1,15] = .752, *P* = .41).

In the condition with eye and mouth motion, cue validity significantly improved reaction times (*F*[1,15] = 4.829, *P* = .04) and there was no main effect of participant group (*F*[1,15] = .115, *P* = .74). However, there was a significant interaction between cue validity and participant group (*F*[1,15] = 11.073, *P* = .005). Simple effects analysis showed that cue validity significantly improved reaction times in the control group (*t*(8) = − 5.361, *P* = .001) but not in the autism group (*t*(7) = .634, *P* = .55).

## 4. Discussion

Gaze cueing using live face-to-face interactions was successful in these children with autism except in the presence of speech utterances. The basic condition, with eye motion, demonstrated gaze cueing in children with autism and in typically developing children. The condition without eye motion also demonstrated gaze cueing in both groups. It would appear that motion was not essential for gaze cueing in these age- and IQ-matched groups of children. Overall we believe there was little evidence that gaze cueing was developmentally delayed in the autism group. This is consistent with previous findings of reflexive orienting to static, averted gaze stimuli in children with autism [13, 14, 16]. 

In the condition with eye motion and speech utterance, gaze cueing was still evident and of the same magnitude in typical children but not in children diagnosed with autism. The simple effect of cue validity was significant only for control participants, and the absence of the gaze cueing effect in the autism group was quite clear (*P* = .55). On average, mouth motion began about 145 ms before gaze shifts began. It can therefore be assumed that mouth movement, or some other aspect of the additional speech cue, abolished the cue validity effect in the autism group in this task. 

It is possible in the static gaze cue condition that participants were able to detect eye motion below the eyelids and this is a potential flaw in the design of the experiment. However, even if this condition is excluded from consideration the fact remains that these children diagnosed with autism demonstrated normal gaze cuing to dynamic gaze cues, and this was completely disrupted when speech utterances were presented simultaneously. 

The small sample size in this study limits the generalizability of these findings, and an extended replication of the findings is warranted. Groups were, however, very closely matched on four variables. It was not possible to have participants in the autism group rediagnosed by a single clinician, which would have been ideal. However, an existing diagnosis was a requirement for all participants with autism. Additionally, data was only analysed for those autism and control group participants who scored within the appropriate range on the ASQ (which is derived from standard assessment measures). 

This study demonstrated that gaze cueing can occur in autism and can be used as a cue for reflexive shifts of attention. This is consistent with a substantial body of evidence from other studies [13, 16]. This study makes the unique contribution of demonstrating this ability using real face-to-face interactions. The susceptibility to interference from mouth movements and speech in the children with autism contrasts with the ability of the general population to covertly, rather than overtly, attend to information from the mouth [2]. The findings imply that children with autism may be unable to perform gaze cueing in typical real-life situations in which speech is a common feature of interactions between children and their peers or carers. The Mindblindness model [1] cannot explain these findings: gaze cueing was neither “intact” nor “impaired” in the autism group. Instead, it is possible that an autism-specific (or individually specific) gaze-following module may have developed in the children with autism in this study. Such atypical development may be one manifestation of a diminished salience of social stimuli in autism. This hypothesis is consistent with atypical neural activity in the fusiform gyrus and amygdala in autism, as observed in response to faces and other visual stimuli (see [22]) and is also consistent with other recent findings of atypical development of gaze direction detection and use of gaze as attention cues in autism [10, 11, 13, 15]. It is also possible that autistics have a specific problem with the prioritisation of cross-modal attention cues, and this could be consistent with their known difficulties with speech perception. Further research will be required to disambiguate these alternative possibilities.

## Figures and Tables

**Table 1:** Matching data for the autism and control groups. Values are mean values ± SD.

	Age (years)	Vocabulary scaled score	Picture completion scaled score	Prorated IQ
Autism group (*n* = 8)	14.3 ± 1.1	11.8 ± 3.5	10.9 ± 3.1	107.6 ± 17.1
Control group (*n* = 9)	13.9 ± 1.2	11.0 ± 3.5	11.0 ± 3.1	105.9 ± 16.8
Group matching	(*t*(15) = .703, *P* = .49)	(*t*(15) = .439, *P* = .67)	(*t*(15) = −.082, *P* = .94)	(*t*(15) = .211, *P* = .84)

**Table 2:** Data on the experimenter's cue durations: cue-target durations, proportions of trials with errors, and *t*-tests for differences in mean cue-target duration for each group.

Condition	Cue-target duration (mean of medians)		Errors	
	Valid cues	Invalid cues	% error trials	Autism versus control trials
With eye motion	400 ms	402 ms	0.9%	(*t*(15) = − 1.775, *P* = .10)
Without eye motion	227 ms	235 ms	7.6%	(*t*(15) = .041, *P* = .97)
With eye and mouth motion	473 ms	473 ms	2.5%	(*t*(15) = − 1.156, *P* = .27)

**Table 3:** Data on participants' reaction times: mean reaction times, proportions of trials with errors, and *t*-tests for differences in mean reaction times between groups.

Condition	Mean reaction time		Errors	
	Valid cues	Invalid cues	% error trials	Autism group versus control group
With eye motion	343.3 ± 56.0 ms SD	376.5 ± 61.8 ms SD	0.3%	*t*(15) = −.497, *P* = .63
Without eye motion	372.9 ± 130.6 ms	401.3 ± 125.3 ms	0.2%	*t*(15) = −1.420, *P* = .18
With eye and mouth motion	345.2 ± 82.3 ms SD	360.0 ± 75.0 ms SD	0.3%	*t*(15) = .716, *P* = .49

**Table 4:** Mean reaction times for the autism and control groups for each condition. Values are in milliseconds (±SD).

Condition	Autism			Control		
	Valid cues	Invalid cues	All trials	Valid cues	Invalid cues	All trials
With eye motion	352.1 ± 68.7 ms	373.1 ± 83.7 ms	363.3 ± 75.5 ms	328.4 ± 42.1 ms	371.0 ± 30.8 ms	350.0 ± 33.7 ms
Without eye motion	341.9 ± 67.6 ms	363.9 ± 69.4 ms	353.1 ± 67.0 ms	351.3 ± 86.3 ms	392.0 ± 96.5 ms	372.0 ± 90.4 ms
With eye and mouth motion	363.0 ± 103.6 ms	356.0 ± 94.6 ms	359.8 ± 98.0 ms	318.9 ± 54.3 ms	351.5 ± 48.8 ms	335.5 ± 50.7 ms
